# GTRF: A Game Theory Approach for Regulating Node Behavior in Real-Time Wireless Sensor Networks

**DOI:** 10.3390/s150612932

**Published:** 2015-06-04

**Authors:** Chi Lin, Guowei Wu, Poria Pirozmand

**Affiliations:** School of Software, Dalian University of Technology, Road No. 8, Development Zone, Dalian 116620, China; E-Mails: wgwdut@dlut.edu.cn (G.W.); pirozmand@mail.dlut.edu.cn (P.P.)

**Keywords:** wireless sensor network, real-time, fault tolerance, selfish behavior, game theory, Nash Equilibrium

## Abstract

The selfish behaviors of nodes (or selfish nodes) cause packet loss, network congestion or even void regions in real-time wireless sensor networks, which greatly decrease the network performance. Previous methods have focused on detecting selfish nodes or avoiding selfish behavior, but little attention has been paid to regulating selfish behavior. In this paper, a Game Theory-based Real-time & Fault-tolerant (GTRF) routing protocol is proposed. GTRF is composed of two stages. In the first stage, a game theory model named VA is developed to regulate nodes’ behaviors and meanwhile balance energy cost. In the second stage, a jumping transmission method is adopted, which ensures that real-time packets can be successfully delivered to the sink before a specific deadline. We prove that GTRF theoretically meets real-time requirements with low energy cost. Finally, extensive simulations are conducted to demonstrate the performance of our scheme. Simulation results show that GTRF not only balances the energy cost of the network, but also prolongs network lifetime.

## 1. Introduction 

Wireless sensor networks (WSNs) are deployed in a variety of environments for reporting periodical events [1,2]. In some cases, especially in emergency or disaster monitoring, real-time and fault-tolerant properties are extremely important because a slight transmission failure may cause unpredictable damages, therefore, it is necessary to guarantee the real-time and fault-tolerant characteristics of WSNs.

In recent years, extensive studies have been carried out on real-time and fault-tolerant routing protocols, which have become one of the most critical research topics in WSNs [3,4,5]. Existing real-time and fault-tolerant routing protocols [6,7,8,9,10] explore location relationships between the current node, neighboring nodes and sink nodes to dynamically find appropriate paths to transmit packets. Although great efforts have been made, the influences of nodes’ behaviors, especially selfish behaviors, cannot be overlooked. In [11], Yang *et al.* envisioned that with the development of artificial intelligence, when nodes became smart enough, they would become capable of controlling their own behaviors. In that case, although a network may be equipped with sound and perfect routing protocols or techniques, which guarantee reliable, real-time and fault tolerant transmissions, if some nodes behave abnormally or selfishly, the whole network will be in danger. 

The side effect of selfish nodes is terrible, especially in real-time transmission. They create void regions and block message transmission [6]. When a selfish node is located in a network, all the messages delivered to it will be dropped due to its selfishness. In fact, the emergence of a node’s selfish behavior arises from pursuing maximum profits such as saving energy to prolong its own lifetime. Commonly, a node with selfish behavior (*i.e.*, a selfish node) only transmits its own sensory data to the next hop, but refuses to relay any packet received from upstream nodes. Although the selfish node saves its own energy, it sacrifices the energy consumption of other nodes, which may eventually shorten the lifetime of the network. 

Therefore, how to regulate the actions of smart nodes, especially their selfish behavior, has become a crucial problem. In the literature, approaches of constraining selfish nodes [11,12,13,14,15] can be categorized into three kinds: selfish behavior precautions [11], selfish node detection [14], and selfish node avoidance [15]. Although many efforts have been made, little literature addresses how to regulate the behavior of sensor nodes, such as developing several rules for selfish nodes to obey. As each node seeks to maximize its own profit, it will spontaneously regulate its behavior by obeying rules. 

Motivated by developing a method for regulating selfish behavior, in this paper, a Game Theory-based Real-time Fault-tolerant (GTRF) protocol is proposed. GTRF consists of two models: (1) a VA model and (2) a jumping transmission model. The VA model is designed to designate cluster heads, achieve fault tolerance, balance the energy cost and regulate the behaviors of cluster members. The jumping transmission model is developed to guarantee the real-time transmission and regulate the behaviors of cluster heads. Moreover, it can avoid the influences of void regions. The contributions of this paper can be summarized as follows:

(1) For ease in controlling nodes’ behaviors, cluster structures are established for grouping nodes into clusters. Therefore, the problem of controlling nodes’ behaviors becomes one of regulating the behaviors of cluster heads and cluster members, respectively. Moreover, a cluster structure is capable of balancing energy consumption in networks.
(2) To regulate nodes’ behaviors within clusters, we develop a VA model, which forces selfish nodes to spontaneously behave as normal nodes so as to maximize their profits. We prove that for selfish nodes behaving as normal nodes is a Nash Equilibrium strategy.
(3) To control nodes’ behaviors among cluster heads, we introduce a jumping transmission model. As selfish nodes are still likely to be selected as cluster heads, in the jumping transmission stage, a feedback mechanism is used which forbids selfish nodes from dropping packets. We also proved that behaving as normal nodes is also a Nash Equilibrium strategy for selfish cluster heads.

The remainder of this paper is organized as follows: Section 2 introduces related work. Section 3 provides the preliminary information necessary for the development of the protocols. Section 4 presents detailed information about GTRF. The performance of GTRF is evaluated in Section 5. Finally, Section 6 concludes the paper and outlines our future works.

## 2. State of the Art

The growing interest in WSNs and the continual emergence of new techniques has inspired some efforts to design reliable routing protocols, and in recent years, more and more researchers have been paying attention to studying real-time routing protocols. In our work, we propose a game theory selfish node prevention protocol for real-time WSNs, hence, in the following we will respectively introduce the state of the art of real-time routing protocols and game theory in the WSN area.

### 2.1. Real-Time Routing Protocols

In general, real-time routing protocols can be categorized into two kinds based on their way of guaranteeing real-time transmission: spatial relation [6,7,8,9] and jumping transmission [10]. With respect to spatial relations [6,7,8,9], the spatial relationship among current nodes, neighboring nodes and the sink node is used to dynamically construct a path to realize real-time transmission. SPEED [6], which estimates the successful transmission rate between the current node and the sink node, is one of the most classical real-time routing protocols for WSNs. It establishes paths which ensure that packets can be sent to the sink node before a predefined deadline. However, when a node failure or a void region [9] are detected, such information cannot be fed back to upstream nodes promptly, which thus affects the subsequent transmissions or even causes packet losses. Two heuristic methods, namely SPEED-T and SPEED-S, which select the next hop according to the minimal transmission delay and fastest transmission speed, respectively, are proposed in [6]. SPEED-T and SPEED-S indeed improve upon the real-time performance of SPEED, but they do not overcome its drawbacks. In reference [7], the range for candidate searching in SPEED is extended to two hops, which enhances the successful transmission rate, however, this approach cannot overcome the drawbacks of SPEED either. A multi-path and multi-level SPEED routing protocol (MMSPEED) is proposed in [8], which dynamically selects the next hop according to the distance between the current node, neighboring nodes and sink node. It establishes the topology according to the reachability of nodes. However, since the reachability is an exponential function of the distance between the current node and the sink node, therefore, this protocol it is not suitable for large-scale networks and long-distance transmissions. A real-time fault tolerant routing protocol called FTSPEED, which is an extension of SPEED, is proposed in [9]. Data can be successfully sent to the destination by bypassing the void region. FTSPEED thus reduces the impact of the void region, but extends the length of paths, which yields a shorter packet remaining time. 

For the jumping transmission protocol, in our previous works we have proposed the DMRF routing protocol for real-time transmission in WSNs [10]. When faulty nodes, network congestion or void regions are encountered, a jumping transmission manner will be utilized. Besides that, when the remaining time ratio decreases to a certain threshold, jumping transmission will also be used, which guarantees that the packets can be successfully delivered to the sink nodes before a predefined deadline. However, energy conservation issues were not mentioned. 

### 2.2. Game Theory in WSNs

In the game theory aspect, although no such research has been proposed to guarantee the real-time performance and fault tolerance of wireless networks, some significant references still deserved to be mentioned. Yang *et al.* focused on the issue of selfish nodes in wireless networks [11]. They summarized and analyzed the typical routing mechanisms in these areas, and systematically categorized the techniques according to their ability to prevent the impact of selfish nodes. Felegyhazi *et al.* proposed a model based on both game theory and graph theory to investigate the Nash Equilibrium conditions of packet forwarding strategies [12]. It utilizes feedback and cooperation mechanisms, which successfully restrict the selfish nodes. Kannan *et al.* developed a game theory-based pure strategy-based aggregation method to achieve reliable transmission [13]. They defined two distinct payoff functions to evaluate the behavior of nodes. Although the existing methods or protocols [14,15,16,17,18,19,20,21,22,23,24,25,26,27,28,29,30,31,32] play important roles in improving the performance of WSNs, the design of real-time fault-tolerant routing protocols is still a challenging issue. In this paper, a game theory-based real-time fault-tolerant routing protocol (GTRF) is proposed to address this problem. 

## 3. Preliminaries

In this section, we describe the related background used in the remaining sections of this paper.

### 3.1. Game Theory and Nash Equilibrium

In this paper, we use game theory to regulate nodes’ behaviors. We firstly give some related game theory definitions. Game theory involves three basic elements, player set, strategy set and payoff function, which are defined as follows:

#### 3.1.1. Player Set ***N***

In this work, we consider that all nodes in the network take parts in the game. Therefore, *N* is denoted as the set of all nodes, $N=\{n_{1},n_{2},...n_{k}\}$. When taking part in a game, each player needs to specify a strategy from its strategy set *Si*. 

#### 3.1.2. Strategy Set

The strategy set *S_i_*, which covers all kinds of strategies $s_{i}$ that user $n_{i}$ may choose, is defined as $S_{i}=\{s_{i}\}=\{s_{i}^{1},s_{i}^{2},...,s_{i}^{n}\}$, where $s_{i}^{j}$ represents the *j*th strategy of node *i*. We denote $s_{-i}$ as the strategy set of other nodes when user $n_{i}$ chooses $s_{i}$. After each node $n_{i}\in N$ determines its strategy $s_{i}\prime$, we define $\Omega \prime =(s_{1}\prime ,s_{2}\prime ,...,s_{n}\prime )$ as a situation that contains every node’s strategy. Moreover, Ω′ can also be expressed as $\Omega \prime =\{s_{i}\prime ,s_{-i}\prime \}$. 

#### 3.1.3. Payoff Function

As every strategy has a one-to-one correspondence with the profit, it is necessary to evaluate the payoff function of a certain node after one strategy is determined. In our game, we denote the payoff function $u_{i}(s_{1}\prime ,s_{2}\prime ,...,s_{n}\prime )$ (or $u_{i}(s_{i}\prime ,s_{-i}\prime )$) as the benefit of node *i* in a certain situation $\left\{s_{1}\prime ,s_{2}\prime ,...,s_{n}\prime \right\}$. Since every node has intelligence, therefore, before choosing a strategy, every node will: (1) evaluate how much profit it will get and then (2) choose a best strategy to maximize the value of $u_{i}(s_{1}\prime ,s_{2}\prime ,...,s_{n}\prime )$.

Then we analyze the status of situation after all nodes have determined their strategies. In our scheme, we use the notion of Nash Equilibrium to regulate the behavior of nodes. Nash Equilibrium [11] is a solution concept of a game involving two or more nodes, in which each node is assumed to know the equilibrium strategies of the other nodes, and no node has anything to gain by changing only its own strategy unilaterally. If each node has chosen a strategy and no node can benefit by changing its strategy while the other nodes keep unchanged, then the current set of strategy choices and the corresponding payoffs constitute Nash Equilibrium. A situation that satisfying Nash Equilibrium can be formalized using Equation (1):

(1) $$\exists s^{*}=\{{s_{1}}^{*},{s_{2}}^{*},...{s_{n}}^{*}\},\;\forall s_{i}\in S_{i}\;\Rightarrow u_{i}({s_{i}}^{*},s_{-i})\geq u_{i}(s_{i},s_{-i})$$

here, $s*=\{{s_{1}}^{*},{s_{2}}^{*},...,{s_{n}}^{*}\}$ is called a *Nash Equilibrium point* and the result of the strategy is considered as a *Nash Equilibrium.*

### 3.2. Auction Bidding Process

In our scheme, a bidding process is used. Hence, we give related definitions. The auction process occurs in a cluster periodically every $T_{period}$ seconds. In the cluster, a cluster head is responsible for managing the execution of the auction. Each time, a node sends a packet that contains a bidding value *bid^i^_Energy_*_(*j*)_ as its *remaining energy* to the cluster head based on its *actual remaining energy bid^i^_basis_*_(*j*)._ At the end of each bidding round, by using specific comparison rules, a winner of the auction in that round will be determined. The winner will be designated as the cluster head in the next cluster period.

In general cases, the value of the remaining energy should equal the actual remaining energy, however, considering that selfish nodes may bid a fake remaining energy value in the bidding process, therefore, it is necessary to avoid such behavior during the bidding process.

### 3.3. Hypotheses and Definitions

In this section, related definitions and hypotheses are given. In GTRF, as the clustering structure is constructed, firstly, we give related definition used within clusters:

**Definition 1.** *Neighboring cluster head: A cluster head that locates in the sensing range of another one*.
**Definition 2.** *Cluster period: The time interval between two continuous cluster head selections*.

Then several hypotheses for supporting GTRF are given. To regulate the nodes’ behaviors especially the selfish behaviors, we have the following hypotheses:

H1 Each node is intelligent to control its behavior and evaluate its profits when choosing a strategy. All nodes are aware and obey the VA model rules, so when selecting their strategy, they always pick out the best choice to obtain maximum benefit. After the cluster structures are established, nodes will periodically send their sensed data to their cluster head. In our scenario, as all nodes are uniformly distributed, the distance between two neighboring nodes are small, and the energy difference between them within a cluster period is trivial, and can therefore be omitted. Therefore, we propose Hypothesis 2.

H2 In a cluster period, each cluster member consumes the same energy amount *f* in communicating with the cluster head. Since a real-time wireless sensor network is required to have fault-tolerant properties, therefore, we propose the following methods to mark whether a node is a faulty node.

H3 A cluster member does not communicate with the cluster head for more than i will be recognized as a faulty node. In GTRF, to avoid selfish behavior, a selfish node will be regarded as a faulty node once it is found. Packets that are sent by faulty nodes will be considered as useless, and they will be dropped immediately, therefore, we propose the following hypotheses:

H4 If the sender of a message is regarded as a faulty node, it will be immediately dropped.

### 3.4. Energy Consumption Model

The energy cost of a sensor mainly consists of sending costs *Cost_S_* and receiving costs *Cost_R_*. Like reference [31], we define the energy consumption functions in transmitting a packet of these two costs using Equations (2) and (3). To send a *k*-bits message at a distance of *d*, a sensor will consume:

(2) $$Cost_{S}=50\;\times \;10^{-9}\;\times \;k+100\;\times \;10^{-12}\;\times \;k\;\times \;d^{2}$$

To receive this message, a sensor will spend:

(3) $$Cost_{R}=50\;\times \;10^{-9}\;\times \;k$$

## 4. GTRF Routing Protocol 

### 4.1. Problem Statement

We consider that a network is composed of *N* nodes, $\;N=\{n_{1},n_{2},...n_{k}\}$. Each node is intelligent and is able to control its behavior. To regulate the behavior of a node, it is necessary to regulate: (1) the action behavior and (2) the bidding behavior. Regulating the action behavior refers to controlling a node’s behavior during data collection, aggregation and forwarding. The action strategies of a node are shown in Table 1.

**Table 1:** The action strategies of a node.

Action	Strategy	Meaning
Sense	{Sense, NSense}	Monitor the environment or not
Communicate (Bid)	{Communicate, NCommunicate}	Communicate with other nodes or not
Forward (Send)	{Send, NSend}	Forward a packet or not

In this paper, we consider a sensor mainly performs three kinds of actions: sense, communicate and forward. With regard to the sensing action, a node is capable of choosing whether to sense the environment or not. For the communication action, it refers to the bidding behavior in the auction. A node can choose whether to communicate with others or not. After a node receives a packet from an upstream node, it can decide whether to forward this packet or not. Based on these definitions, our problem can be summarized as follows:

1. **Input:** Network parameters, strategy set (*i.e.*, Action strategy and bidding strategy).
2. **Find**: Strategies $s_{i}*$for node i in different situations.
3. **Such that**: $\forall \Omega \prime =\{s_{i}\prime ,s_{-i}\prime \}$, $u(s_{i}*,s_{-i})$ is the maximum.
4. **Meanwhile**: $s_{i}*$ is a Nash Equilibrium point and the corresponding situation reaches Nash Equilibrium.

As shown in the problem formalization, if we can satisfy the objective of the problem, which means in any situation: (1) the strategy of a node is a best choice; (2) all the strategy constitutes a Nash Equilibrium point and (3) the situation is a Nash Equilibrium, we can successfully regulate the behavior of any node, no matter whether a node is selfish or not. 

Therefore, in the following section, we propose a game theory-based selfish preventative routing protocol to regulate the behaviors of all nodes and simultaneously guarantee the network’s real-time and fault-tolerant features. 

### 4.2. GTRF Overview 

**Figure 1:**
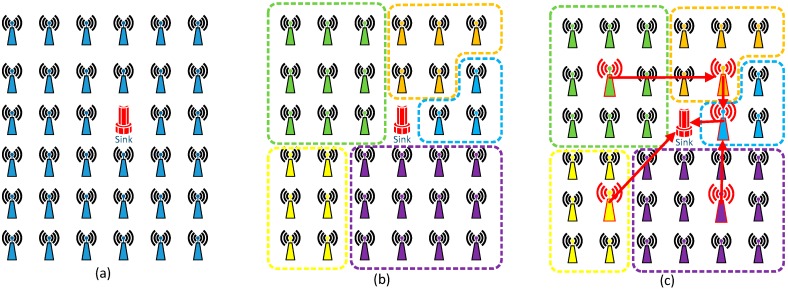
An overview of GTRF. (**a**) Nodes are uniformly distributed; (**b**) Clustering protocol is applied, which groups all nodes into five clusters; (**c**) Each cluster designate a cluster head for data collecting, aggregating and transmitting.

In this section, we give detailed information about the GTRF protocol. GTRF is composed of two parts: a VA model and a jumping transmission model. The architecture of GTRF is shown in Figure 1, where originally, all nodes are uniformly deployed in the network (see Figure 1a), and then a clustering protocol FATP [26] is utilized to group all nodes into clusters (see Figure 1b); in the figure, five clusters are established. The reason why we used FATP is that FATP can generate clusters of nearly equal size, which is useful for balancing the energy cost between clusters. In each cluster, a cluster head is designated (see Figure 1c), which is responsible for managing: (1) the bidding process and (2) the cluster head selection in the next round. 

### 4.3. VA Model

After the cluster structure is constructed, all nodes will be grouped into clusters. In a cluster, a cluster head will be selected to manage the working process (*i.e.*, data transmission and bidding) of all nodes that belong to that cluster. In our scheme, the TMDA MAC protocol is used for assigning time slots for all nodes within a cluster, which is useful for avoiding wireless medium access conflicts. 

**Figure 2:**
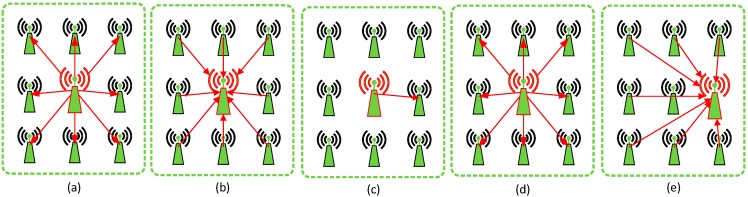
The VA model process. (**a**) The cluster head notifies the beginning of an auction; (**b**) Each node bids a value based on its remaining energy; (**c**) The winner of the auction is designated as the cluster head in the next round, and related information is sent from the original cluster to it; (**d**) The original cluster head broadcasts the result of the auction; (**e**) The auction process ends and the working process begins—cluster members transmit their data to the new cluster head.

Each node monitors the environment, generates sensory data, and sends it to the cluster head in its pre-assigned time slot. Then data will be collected and aggregated by the cluster head, and finally, data will be forwarded to other neighboring cluster heads. When a selfish node exists in a cluster, for the sake of saving energy, it will not monitor the environment or send its sensory data to the cluster head. To avoid such a phenomenon, we develop a bidding model, which is similar to Vickrey Auction [27] used in the economic research field, therefore, we name it VA model. The VA model aims to limit the behavior of the nodes in the cluster, which ultimately yields a selfish node working normally like normal nodes. In fact, the VA model is an auction process, and each time, every node bids an energy value as its remaining energy. The process of the VA model is illustrated in Figure 2. In the VA model, four steps and three rules are designed. After a cluster is established, a cluster head will be designated and then all nodes start working. Each node will periodically send data to the cluster head. As cluster heads suffer from a larger energy consumption than cluster members, to alleviate the energy consumption imbalance, a cluster head should be re-elected. Then a new cluster head will be designated to manage the cluster. In the following, we describe the steps and rules in detail:

*Step* *1* The cluster head broadcasts a message to notify the beginning of the bidding process (see Figure 2a).
*Step* *2* After receiving the notification message, the bidding process begins. Each node (including the cluster head and all cluster members) determines a bidding value as its remaining energy. When calculating the bidding value, each node should refer to its actual remaining energy so as to bid a proper value. However, this does not mean the bidding value should be exactly the same as the actual remaining energy.
*Step* *3* Each node sends its bidding value to the cluster head in its pre-allocated time slot (see Figure 2b). The cluster head records all bidding values so as to select a new cluster head in the next round.
*Step* *4* The cluster head selects the node whose bidding value is the biggest as the cluster head in the next cluster period (see Figure 2c). Then the cluster will broadcast this information to all nodes within the cluster (see Figure 2d). If the newly selected node is a cluster member, the cluster head will transmit information such as TMDA assignments, node ids and so on to the newly selected node. In the next cluster period, the original cluster head will change its role into a cluster member. Finally, a new cluster head is designated, and the working process begins, each node sends its data to the newly selected cluster head (see Figure 2e). The cluster head collects and aggregates the data and then transmits it to other cluster heads.

In order to successfully proceed with the auction, three rules are given that each node must obey. To determine which node will win the auction and will be designated as the cluster head in the next cluster period, we propose Rule 1.

*Rule* *1* A node bidding the highest *bid^i^_Energy_*_(*j*)_ is regarded to win the auction and it will be designated as the cluster head in the next cluster period. The energy cost of a cluster head is mainly consumed in four actions: sensing energy cost, packet receiving cost, data processing cost and data sending cost. The energy cost of a cluster head is much bigger than that of a cluster member. Therefore, we need to control the energy cost of the cluster head so as to avoid creating big energy consumption gaps between a cluster head and its cluster members.
*Rule* *2* A cluster head consumes at most g(i)energy in the *i*th cluster period. With respect to a cluster member, we also propose a rule for limiting the energy cost.
*Rule* *3* A cluster member consumes more than *f* will stop working in that cluster period. In the VA model, each node is aware of the rules and must obey them. Since each node seeks the maximum value of the payoff function, it will firstly estimate the profit and then bid *bid^i^_Energy_*_(*j*)_. Based on these rules, we prove that, although one or more selfish nodes may exist in a cluster, the best strategy for a cluster member is to behave like a normal node (see Theorem 4 in Section 4.5).

Besides that, we propose Rule 4 for judging a faulty node. Intuitively, if a node rejects doing anything and remains idle, it will save lots of energy. In that case, according to Hypotheses 3 and 4, it will be regarded as a faulty node. All the messages sent from it will be dropped. In that case, that node will be forever isolated from the network. This is not a best choice but a worst choice, because from then on, all the packets sent from it will be dropped and no nodes will ever communicate with it. Therefore, rejecting doing anything (*i.e.*, sensing, collecting, forwarding) for a node is not a best choice.

*Rule 4*. The profit that a node regarded as a faulty node is the minimum.

### 4.4. Jumping Transmission Model

In Section 4.3, we mainly studied how to regulate the selfish behavior within clusters. However, only constraining the behaviors in a cluster is not sufficient, because a selfish node could still be selected as a cluster head. In that case, suppose the selfish cluster head drops all received packets from other neighboring cluster heads, then the real-time transmission process will be destroyed. Therefore, it is necessary to regulate the behavior of cluster heads. To avoid the destruction caused by selfish cluster heads, we propose a jumping transmission model, which is similar to reference [10]. In our scheme, all nodes are supported by short and long range transmission.

The jumping transmission model consists of the jumping forwarding model and feedback message notification model, which are described in Algorithms 1 and 2, respectively. As shown in Algorithm 1, in the jumping forwarding model, when a cluster head $C_{i}$ tends to forward a packet downstream, it will first determine whether the jumping transmission is applied or not. Once network congestion, faulty nodes or void region are detected or the remaining time ratio [10] of the message is falls below the threshold (*i.e.*, $\text{λ}<{\text{θ}}_{\text{jump}}$), the transmission mode will switch to jumping transmission mode. Cluster head $C_{i}$ will select a downstream cluster head $C_{j}$ that is outside the transmission range (detailed information is given in reference [10]), and then $C_{i}$ directly sends *p* to $C_{j}$. The jumping transmission is able to reduce the transmission delay and guarantee that data packets can be sent to the destination node before the specified time deadline. When there is no need to use the jumping transmission model, $C_{i}$ selects a downstream cluster head $C_{j}$ that is inside the transmission range, and send p to $C_{j}$ in a hop-by-hop manner.

In Algorithm 2, the feedback message notification model is given. When the sink node receives a packet *p* from $C_{i}$, it will broadcast to notify $C_{i}$ that packet p has been successfully received. Then $C_{i}$ will wait for the notification message. If $C_{i}$ didn’t get the reply from *Sink* for φ time, which means there must be a selfish node within the path from $C_{i}$ to *Sink*. Then $C_{i}$ will directly send all packets to *Sink* and broadcast that all cluster heads within the path from $C_{i}$ to *Sink* are faulty nodes.

Based on the jumping transmission model, we can regulate the behavior of selfish cluster heads, as proved in a later section.

**Algorithm 1** Jumping forwarding model1:Network initialization2:Cluster construction and initialization3:Establish routing tables for all clusters $C_{i}\in C$4:**If**
$C_{i}$ intends to transmit a packet p
**then**5:  **If** (${\text{λ < θ}}_{\text{jump}}$) || (congestion, faulty nodes or void region are detected ) **then**6:   Select a downstream cluster head $C_{j}$ that is outside the transmission range (detailed information is given in reference [10])7:   Use the jumping transmission method to send p from $C_{i}$ to $C_{j}$8:  **Else**9:   Select a downstream cluster head $C_{j}$ that is inside the transmission range10:   Use the hop-by-hop transmission method to send p from $C_{i}$ to $C_{j}$11:  **Endif**12:**Endif**13:Adjust transmitting probabilities according to reference [10]

**Algorithm 2** Feedback message notification model1:Cluster head $C_{i}$ sends a packet p to the *Sink*2:**If** the *Sink* receives a packet p orignated from cluster head $C_{i}$
**then**3:  The sink will broadcast to notify $C_{i}$ that packet p has been successfully sent to *Sink*4:**Endif**5:**If**
$C_{i}$ didn’t get the reply from *Sink* for φ time **then**6:  $C_{i}$ will from now on directly deliver all packets to *Sink*7:
$C_{i}$ will broadcast that all clusters heads within $C_{i}$ to *Sink* are all faulty nodes7**Endif**


### 4.5. Characteristics Analysis

In this section, the characteristics of GTRF are analyzed in detail. To highlight the advantages of GTRF in terms of energy cost, lifetime and so on, several theorems have been proposed and proved. The core of the VA model is a bidding process, whereby each time a node needs to bid a value based on its actual remaining energy. A node with the biggest value will be designated as a cluster head in the next cluster period. As each node is intelligent to control its behavior, it can decide to bid any value for obtaining the maximum profit. Therefore, Theorem 1 is proposed which aims at proving that GTRF is able to regulate selfish behaviors within clusters. By using GTRF, a selfish node can only bid its actual remaining energy.

In the bidding process, we denote *bid^i^_Energy_*_(*j*)_ as the bidding value that node *i* bids in the *j*th bidding round. At the same time, *bid^i^_basis_*_(*j*)_ is denoted as the actual remaining energy of node *i* in the *j*th bidding round. As the second highest bidding value is an important factor that determine which node will win, therefore, we defined *bid_second_*_(*j*)_ as the second highest value in the *j*th bidding round. 

**Theorem 1.** *The best strategy for a node in the VA model is to bid its actual remaining energy (i.e., bid^i^_Energy(j)_ = bid^i^_basis(j)_), the situation including all nodes’ bidding values in one auction round is a Nash Equilibrium when requirements (1) and (2) are satisfied.*

(1) The energy cost of cluster $C_{k}$’s cluster head in the *j*th round is inversely proportional to the average bidding value within $C_{k}$, (*i.e.*, $\frac{1}{g(j)}\propto \frac{1}{\left|C_{k}\right|}\sum_{i≺C_{k}}bid_{Energy(j)}^{i}$ ), where $\left|C_{k}\right|$ is the size of the cluster, $i≺C_{k}$ means node *i* belongs to cluster $C_{k}$.

(2) $T_{period}<\frac{bid_{Energy(j)}^{i}}{\sigma_{c}}-\frac{f}{\sigma_{N}}$ and $\frac{\alpha }{\frac{1}{\left|C_{k}\right|}\sum_{n_{i}≺C_{k}}bid_{Energy(j)}^{i}}<g(j)<bid_{Energy(j)}^{i}$, where α is a constant, $\sigma_{c}$ are $\sigma_{N}$ denoted as the energy consumption speed of a cluster head and a cluster member, respectively.

**Figure 3:**
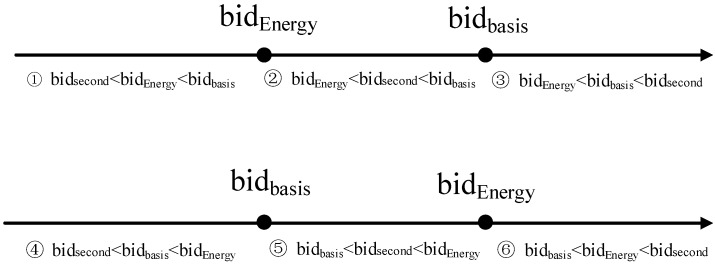
The comparison relationship among $bid_{Energy(j)}^{i}$, $bid_{basis(j)}^{i}$ and $bid_{\sec ond(j)}$.

**Proof.** As shown in Figure 3, when considering the comparison relationship among $bid_{Energy(j)}^{i}$, $bid_{basis(j)}^{i}$ and $bid_{\sec ond(j)}$ there are six situations, as shown in Figure 3.

In scenario ①, where $bid_{\sec ond(j)}<bid_{Energy(j)}^{i}<bid_{basis(j)}^{i}$, node *i*’s biding energy is bigger than the second highest bidding energy $bid_{\sec ond(j)}$(*i.e.*, $bid_{Energy(j)}^{i}$ is the highest), according to Rule 1, node *i* will be eventually selected as the cluster head. As we have $\frac{1}{g(j)}\propto \frac{1}{\left|C_{k}\right|}\sum_{i≺C_{k}}bid_{Energy(j)}^{i}$, if *i* bids a lower value, that will lead to smaller value of $\frac{1}{\left|C_{k}\right|}\sum_{i≺C_{k}}bid_{Energy(j)}^{i}$, which will increase the energy cost in the *j*th round after node *i* is selected as a cluster head. On the contrary, bidding the actual energy will lead to a smaller g(j). Therefore, the strategy of bidding the actual remaining energy is better than bidding a lower value. Thus, $u(bid_{Energy(j)}^{i},s_{-i})<u(bid_{basis(j)}^{i},s_{-i})$ can be drawn. 

In scenario ②, where $bid_{Energy(j)}^{i}<bid_{\sec ond(j)}<bid_{basis(j)}^{i}$, the node bidding $bid_{Energy(j)}^{i}$ will become a cluster member. In contrast, if it bids $bid_{basis(j)}^{i}$, it will become a cluster head. According to *H3*, if a cluster member does not communicate with the cluster head for more than $T_{period}$, it will be considered a faulty node, Therefore, we regard that the cluster member will become a faulty node at the end of cluster period, which means $\frac{g(j)}{\sigma_{c}}-T_{period}\geq \frac{f}{\sigma_{N}}$, therefore we have $T_{period}\leq \frac{g(j)}{\sigma_{c}}-\frac{f}{\sigma_{N}}$. Since $\frac{1}{g(j)}\propto \frac{1}{\left|C_{k}\right|}\sum_{i≺C_{k}}bid_{Energy(j)}^{i}$, node *i* will become a cluster head, which means $bid_{Energy(j)}^{i}>\frac{1}{\left|C_{k}\right|}\sum_{i≺C_{k}}bid_{Energy(j)}^{i}$, meanwhile, according to the definition of g(j), we can draw the same conclusion as (2): $T_{period}<\frac{bid_{Energy(j)}^{i}}{\sigma_{c}}-\frac{f}{\sigma_{N}}$ and $\frac{\alpha }{\frac{1}{\left|C_{k}\right|}\sum_{i≺C_{k}}bid_{Energy(j)}^{\left(i\right)}}<g(j)<bid_{Energy(j)}^{i}$, where α is a constant. Hence, we conclude that becoming a cluster head will produce more benefits than becoming a cluster member. As a result, we have $u(bid_{Energy(j)}^{i},s_{-i})<u(bid_{basis(j)}^{i},s_{-i})$.

In scenario ③, where $bid_{Energy(j)}^{i}<bid_{basis(j)}^{i}<bid_{\sec ond(j)}$, if one or more nodes whose bidding values are bigger than $bid_{Energy(j)}^{i}$ and $bid_{basis(j)}^{i}$, node *i* will eventually become a cluster member. Since its bidding value is bigger than $bid_{basis(j)}^{i}$, which means at the end of the cluster period, node *i* will spend f energy at most. According to *H2*, the cluster will consume identical energy no matter what it bids. Therefore, we have $u(bid_{Energy(j)}^{i},s_{-i})=u(bid_{basis(j)}^{i},s_{-i})$. 

In scenario ④, where $bid_{\sec ond(j)}<bid_{basis(j)}^{i}<bid_{Energy(j)}^{i}$, node *i* will become the cluster head. For$bid_{Energy(j)}^{i}>bid_{basis(j)}^{i}$, meaning that in the worst case, node *i* may consume more energy than g(j). Thus, selecting $bid_{Energy(j)}^{i}$ cannot ensure the continuous operation to the end of the cluster period, Therefore, bidding $bid_{Energy(j)}^{i}$ is worse than bidding $bid_{basis(j)}^{i}$, so we have $u(bid_{Energy(j)}^{i},s_{-i})<u(bid_{basis(j)}^{i},s_{-i})$.

In scenario ⑤, where $bid_{basis(j)}^{i}<bid_{\sec ond(j)}<bid_{Energy(j)}^{i}$, a node bidding its actual energy will become a cluster member, whereas if it bids a bigger value, it will be eventually selected as a cluster head. The energy cost of being a cluster head is much bigger than being a cluster member. Therefore, we can get $u(bid_{Energy(j)}^{i},s_{-i})<u(bid_{basis(j)}^{i},s_{-i})$.

In scenario ⑥, where $bid_{basis(j)}^{i}<bid_{Energy(j)}^{i}<bid_{\sec ond(j)}$, node *i* will become a cluster member. Because $bid_{Energy(j)}^{i}$ is bigger than $bid_{basis(j)}^{i}$ which implies that node *i* may not be able to survive at the end of the cluster period, it is predictable that node *i* will firstly consume $bid_{basis(j)}^{i}$ energy and then stop working, but there still exists the fact that the cluster head will firstly consume g(j) energy , and initiate the next bidding round, in that case, the value of $bid_{basis(j)}^{i}$ will become useless to the bidding results. Therefore, we can draw the conclusion that $u(bid_{Energy(j)}^{i},s_{-i})<u(bid_{basis(j)}^{i},s_{-i})$.

As a result, we conclude that, to maximize the profit, the best strategy is to bid the actual energy. Meanwhile $s*=bid_{basis(j)}^{i}$ is a Nash Equilibrium point and situation of bidding strategies in the auction is a Nash Equilibrium. Theorem 1 guarantees that no matter a node is selfish or not, in the VA model, the behavior of that node can be regulated. Then we analyze how to constrain a node’s behavior in communication action.

**Theorem 2.** *The best strategy for a cluster member is to transmit periodically to the cluster head.*

**Proof.** In the cluster, the strategy set of each node after receiving a message can be generalized as {*SEND*, *NSEND*}. *SEND* and *NSEND* stand for a node’s strategies for whether to send a packet or not. According to Theorem 1, each node will bid its actual energy. If a node rejects sending messages to save energy, its remaining energy will be more than that of others. In that case, two conditions may happen: (1) it will be considered a faulty node; or (2) by referring to *Rule 1*, it will inevitably be assigned as a cluster head which will lead to higher energy consumption. Therefore, the sending strategy *SEND* is better, we have *u*(*SEND*,*s*_‒*i*_) > *u*(*NSEND*,*s*_‒*i*_). Therefore, we can conclude that the best strategy for a cluster member is to transmit periodically to the cluster head. 

From Theorem 1 to Theorem 2, we conclude that the best strategy for a selfish node within a cluster is to behave like the normal nodes. Thus, the selfish nodes will not affect anything in clusters. Another advantage of the VA model is the fault tolerance aspect. Each cluster head periodically broadcasts “Hello” messages to cluster members. After receiving such message, each cluster member will respond to the cluster head in its pre-allocated TDMA time slot. If a cluster head does not receive any reply from a cluster member during the pre-allocated time slot, that node will be recognized as a faulty node. Therefore, all faulty nodes in clusters will be discovered. Nevertheless, the VA model can only regulate the behaviors within cluster, it cannot control unexpected behaviors after a selfish is designated as a cluster head. In that case, a cluster head can drop all packets received from others. Therefore, we propose the following theorem:

**Theorem 3.** *The best strategy for a cluster head is to forward all received packets.*

**Proof.** Suppose a selfish node is designated as a cluster head. To save energy, it will selectively forward parts of packets or totally drop all of them. If packets are dropped, the original sender will broadcast a packet to alert others that there exist selfish nodes in that path. Then the selfish node will be treated as a faulty node (see Algorithm 2). According to *Rule 4*, to avoid getting minimum profits, the selfish node has to forward all packets. Therefore, we can conclude that the best strategy for a cluster head is to forward all received packets. By combining Theorems 1–3, we deduce Theorem 4:

**Theorem 4.** *The best strategy for a selfish node is to act as a normal node all the time in GTRF.*

**Proof.** We discuss this problem by analyzing that a selfish node acts as a cluster member or a cluster head respectively. 

(1) If a selfish node acts as a cluster member, according to Theorem 2, the selfish node has to periodically communicate with the cluster head. Moreover, Theorem 1 restricts each node to bid its actual energy, and that process is proved to reach a Nash Equilibrium. Besides that, we should also consider the case that a node refuses to communicate with any node so as to save energy. According to *Rule 3*, if the node does not communicate with the cluster head for a period of time, it will be considered as a faulty node. Therefore, the selfish node has to act as a normal node. 

(2) If a selfish node becomes a cluster head, according to Theorem 3, it has to forward all received packets just like a normal cluster head does. As a result, we can conclude that selfish nodes have to act like the normal nodes in GTRF. After proving that GTRF is able to regulate the selfish behaviors of nodes, then we analyze the real-time aspect features.

**Theorem 5.** *The GTRF protocol is able to meet the real-time requirements of packets.*

**Proof.** The only differences between the Dynamical juMping Real-time Fault-tolerant routing protocol (DMRF) [10] and GTRF is the time delay, GTRF runs based upon the cluster topology, which saves a lot of energy but prolongs the time of transmission. The principles of the jumping mode in both DMRF and GTRF are identical. Therefore, the jumping manner will be performed when the remaining time factor [10] decreases to a threshold or a void region is detected. The delay of data fusion can only lead to the earlier use of the jumping manner. Therefore, GTRF can still achieve real-time transmission.Besides an analysis on node behavior regulations and real-time features, we also study the lifetime of the network.

To study the lifetime of the network, we firstly define $\frac{1}{g(j)}=\frac{\text{λ}}{\left|C_{k}\right|}\sum_{i≺C_{k}}bid_{Energy(j)}^{\left(i\right)}$, where λ is a variable, satisfying the condition $|1-\text{λ}|<10^{-6}$. 

**Theorem 6.** *The lifetime of the cluster, which is directly relevant to variable λ, meets the condition* $LifeTime>\frac{\lambda }{1\;-\;\lambda }\times T_{period}$*.*

**Proof.** During the *i*th cluster period, every cluster member spends *f* energy in communicating and each cluster head consumes g(i) energy. Without loss of generality, we assume a cluster consists of *k* nodes. In the first round, the *i*th node becomes the cluster head. The remaining energy of each node after the first round $E_{1}^{\left(i\right)}$ will be:

$$\begin{array}{c} E_{1}^{\left(1\right)}=E_{initial}-f\\ E_{1}^{\left(2\right)}=E_{initial}-f\\ ...\\ \begin{array}{l} \;E_{1}^{\left(i\right)}=E_{initial}-g(1)\\ \;...\\ \;E_{1}^{\left(k\right)}=E_{initial}-f \end{array} \end{array}$$

In the *n*th round, we have:

(4) $$\begin{array}{c} E_{n}^{\left(1\right)}=E_{initial}-m_{1}f-\alpha_{1}^{\left(1\right)}g(1)-\alpha_{2}^{\left(1\right)}g(2)-...-\alpha_{n}^{\left(1\right)}g(n)\\ E_{n}^{\left(2\right)}=E_{initial}-m_{2}f-\alpha_{1}^{\left(2\right)}g(1)-\alpha_{2}^{\left(2\right)}g(2)-...-\alpha_{n}^{\left(2\right)}g(n)\\ ......\\ E_{n}^{\left(k\right)}=E_{initial}-m_{k}f-\alpha_{1}^{\left(k\right)}g(1)-\alpha_{2}^{\left(k\right)}g(2)-...-\alpha_{n}^{\left(k\right)}g(n) \end{array}$$

In each round only one cluster is selected, therefore coefficients$\;\alpha_{i}^{\left(j\right)}$ satisfy:

(5) $$\sum_{1}^{k}\alpha_{1}^{\left(i\right)}=\sum_{1}^{k}\alpha_{2}^{\left(i\right)}=...=\sum_{1}^{k}\alpha_{n}^{\left(i\right)}$$

Since a cluster cannot survive until the remaining energy of the cluster head is smaller than the energy cost in the next round, therefore, we have:

(6) $$\frac{\lambda }{k}\sum_{i=1}^{k}E_{n}^{\left(i\right)}\leq g(n+1)$$

By merging Equations (4) and (5), the coefficients $\alpha_{j}^{\left(i\right)}$ and $m_{j}$ can be removed, we can get:

(7) $$\sum_{i=1}^{k}E_{n}^{\left(i\right)}=nE_{init}-n(k-1)f-\sum_{j=1}^{n}g(j)$$

Then, we analyze the relationship between g(i) and g(i+1) :

(8) $$\begin{array}{l} g(1)-\text{g}(0)=-\frac{\lambda }{k}[(k-1)f-g(0)]\\ g(2)-g(1)=-\frac{\lambda }{k}[(k-1)f-g(1)]\\ ...\\ g(n)-g(n-1)=-\frac{\lambda }{k}[(k-1)\text{f}-g(n-1)]\\ g(n+1)-g(n)=-\frac{\lambda }{k}[(k-1)f-g(n)] \end{array}$$

We deduce that:

(9) $$\begin{array}{l} g(1)-g(0)=-\frac{\lambda }{k}[(k-1)f-g(0)]\\ g(2)-g(1)=-\frac{\lambda }{k}[(k-1)f-g(1)]\\ ...\\ g(n)-g(n-1)=-\frac{\lambda }{k}[(k-1)f-g(n-1)]\\ g(n+1)-g(n)=-\frac{\lambda }{k}[(k-1)f-g(n)] \end{array}$$

From Equations (7) and (9), we can get:

(10) $$nE_{initial}-n(k-1)f-\sum_{j=1}^{n}g(j)\leq \lambda E_{initial}-\frac{\lambda }{k}[(n+1)(k-1)f]+\frac{\lambda }{k}\sum_{j=1}^{n}g(j)$$

According to the energy consumption relationship betweeng(i) and f, Equation (11) can be obtained.

(11) $$g(j)<\lambda (E_{initial}-kf)$$

Thus, Equation (12) can be concluded:

(12) $$\frac{\left(n-\lambda )E_{initial}-n(k-1)f+\frac{\lambda }{k}[(n+1)(k-1)f\right]}{\left(1+\frac{\lambda }{k}\right)}\leq \lambda nE_{initial}-\lambda kfn$$

At last, we have:

(13) $$n>\frac{\lambda E_{\mathrm{int}ial}}{(1-\lambda )E_{initial}}=\frac{\lambda }{1-\lambda }$$

Therefore, the $LifeTime=n\times T_{period}>\frac{\lambda }{1-\lambda }\times T_{period}$ can be drawn. Therefore, the lifetime of the network is directly relevant to variable λ.

## 5. Simulation Analysis

In this section, extensive simulations are conducted to show the performance of the proposed scheme. In our simulations, 400 nodes are uniformly deployed in a 20 m × 20 m area. To show the advantages of the proposed scheme, we implement DMRF [10], which is a jumping transmission routing protocol in WSNs. As GTRF is executed on the cluster structure, therefore, we additionally use the classic clustering protocol LEACH to form clusters and then execute DMRF on the cluster structure DMRF + LEACH. Besides that, to show the influences of selfish nodes, we randomly select ten percent of nodes as selfish nodes. Moreover, to highlight the performance of GTRF in real-time transmission, several real-time routing protocols, such as SPEED [6], MMSPEED [8], FTSPEED [9] are implemented. Related parameters are listed in Table 2. In the simulations, all the nodes have been implemented with short and long range transmission capacity, which are indispensable for realizing the jumping transmission. 

**Table 2:** Simulation parameters.

Item	Parameters
Routing Protocol	SPEED [6], SPEED-T [6], SPEED-S [6], MMSPEED [8], FTSPEED [9], DMRF [10], GTRF, DMRF Cluster (DMRF + LEACH)
MAC Protocol	TDMA
Bandwidth	10 Kb/s
Data Packet Size	1024 bit
Region Size	(20 m, 20 m)
Node Number	400, 1000, 2000, 5000, 10,000
Node Distribution	Uniform
TDMA Time Slot	120 ms
Cluster Member Sensing Range	2 m
Cluster Head Sensing Range	5 m
Initial Node Energy	100 J
Selfish Node Ratio	10%

### 5.1. Energy Cost Distribution

**Figure 4:**
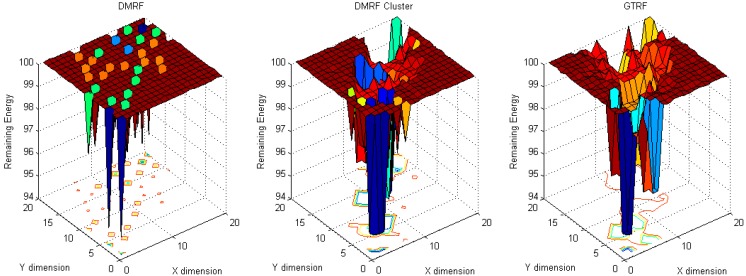
Energy cost with fixed source and fixed sink with selfish nodes.

To analyze the energy cost distribution of the network, in our simulation, one million packets are generated and sent from source nodes to the sink node. To analyze the energy cost distribution, two scenarios: (1) fixed source with fixed sink and (2) random sources with fixed sink, are implemented. Fixed source node refers to the source node located at (20, 20) and the sink node located at (1, 1), whereas in random source nodes with fixed sink, the sink node is located at (10, 10), and sources locate everywhere in the network. From Figure 4 to Figure 7, in each figure, dimension X and dimension Y represent the coordinates of nodes, while the dimension Z stands for the remaining energy of each node.

#### 5.1.1. Fixed Sources with Fixed Sink

**Figure 5:**
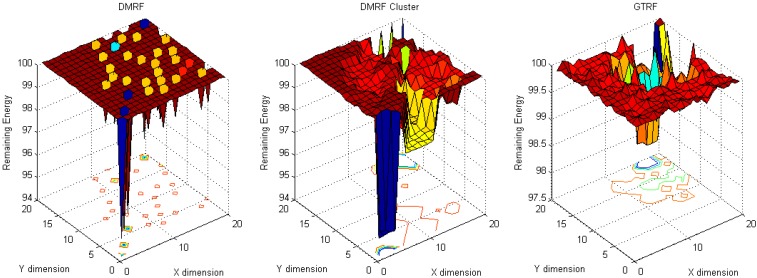
Energy cost with fixed source and fixed sink without selfish nodes.

When the source node and the sink node are fixed, the energy cost distribution results are shown in Figure 4 and Figure 5.

In Figure 4, ten percent of the nodes are selfish nodes. Since a selfish node may discard packets, in DMRF [10], they will be regarded as faulty nodes. DMRF will thereby regard the locations of the selfish nodes as void regions, therefore, it will adopt a jumping transmission manner to forward packets. However, the energy cost is concentrated in a small number of nodes. The reason is that there are some nodes that always appear in others nodes’ forwarding candidate sets [8], therefore, these nodes have higher probabilities of receiving and forwarding packets from others, which leads to higher energy cost. We note that the maximum energy cost is 5.6 J.

To balance the energy cost, we implement the DMRF with LEACH clustering protocol. In that case, due to frequently changing cluster heads, the energy concentration issue is relieved. However, selfish nodes still have the probability of becoming the cluster heads. We note that the maximum energy cost is 5.4 J.

In GTRF, as all the selfish nodes will be regulated, therefore, the maximum energy cost is smaller than those of DMRF and DMRF + LEACH, we note that the maximum energy cost is 4.5 J. Therefore, we can conclude that GTRF can effectively save the energy cost. 

In Figure 5, we analyze the same scenario as Figure 4 but without selfish nodes. Compared with Figure 4, DMRF and DMRF + LEACH achieve more balanced energy cost distributions. However, the maximum energy costs do not change much. In GTRF, the maximum energy cost is only 1.5 J, which is nearly 1/3 of that in Figure 4. As a result, we can also conclude that, GTRF can save energy efficiently.

#### 5.1.2. Fixed Sink with Random Source

**Figure 6:**
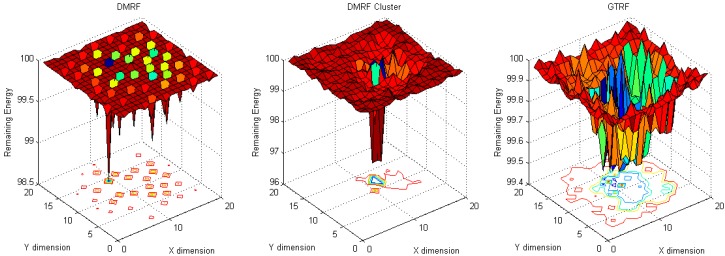
Energy cost with fixed sink with random source with selfish nodes.

In this experiment, the locations of the source nodes are randomized, whereas the sink stays in the center of the region. As shown in Figure 6, in DMRF, normal nodes suffer from more energy cost because the selfish nodes are considered as faulty nodes. 

In DMRF + LEACH, since each cluster head is selected based on probabilities, each selfish node has the probability of becoming a cluster head, which prevents the forwarding process. Therefore, the jumping manner is used frequently which burdens the energy cost of the sink node and its surroundings. Hence, a higher energy cost occurs in the center of the network. In GTRF, we note that the energy cost is much lower than in the other two methods, and the maximum cost node only consumes 0.6 J energy.

**Figure 7:**
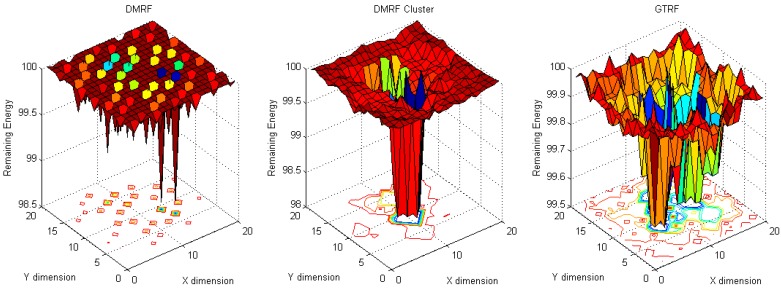
Energy cost with fixed sink with random source without selfish nodes.

In Figure 7, the maximum cost of DMRF is 1.3 J. When utilizing the DMRF + LEACH, we note that the energy cost, which is nearly 2 J, centralizes on the nodes that are nearby the sink node. In contrast to these two schemes, GTRF designates the node with the highest energy to be the cluster head in the next cluster period, which can balance the energy cost. The node with the highest energy cost only consumes 0.6 J to forward the packets. Therefore, GTRF can effectively avoid the influences of selfish nodes and save energy.

#### 5.1.3. Energy Difference between Maximum Cost and Minimum Cost

**Figure 8:**
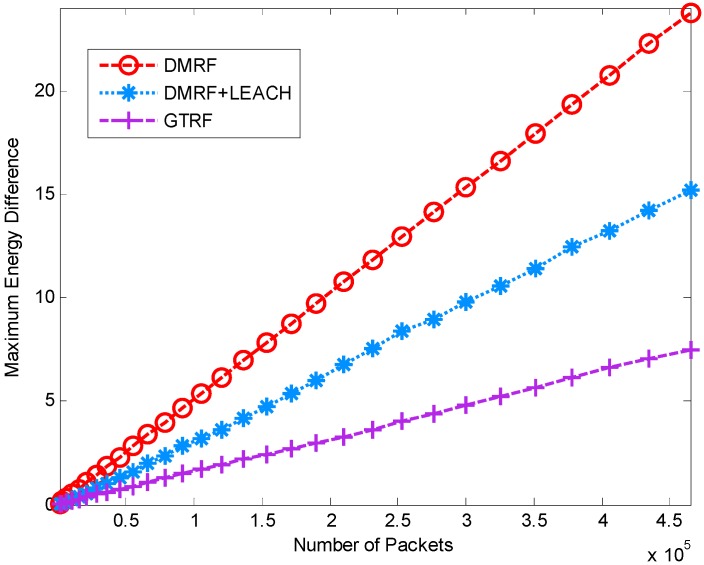
Energy difference between maximum and minimum energy node with selfish nodes.

**Figure 9:**
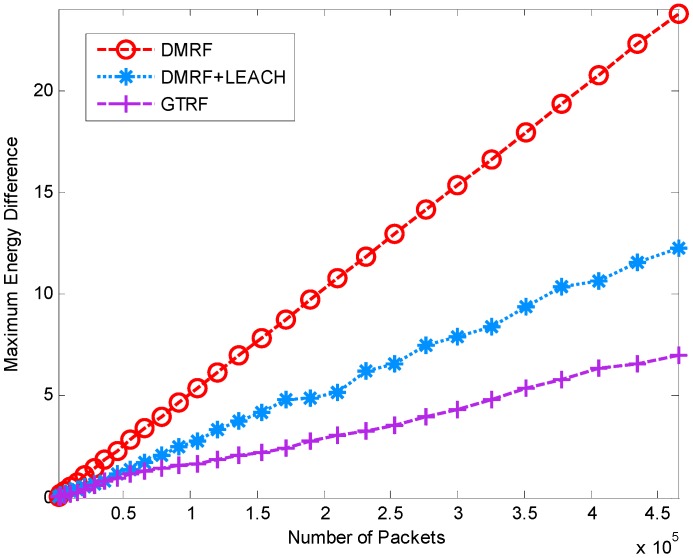
Energy difference between maximum and minimum energy node without selfish nodes.

In this simulation, we investigate the energy difference of different schemes, which is defined as the maximum cost minus the minimum cost in the network. The simulation parameters are identical as in Section 5.1.1. In the simulation, we also discuss the influence of the selfish nodes. Ten percent of nodes are randomly selected as selfish nodes. The results are shown in Figure 8 and Figure 9. 

In Figure 8, the energy differences of the three methods rise smoothly with the increasing initial energy of nodes. GTRF is much lower than the other two methods. DMRF presents a linear incremental fashion. The reason is that some of the nodes always exist in the forwarding candidate sets, which may lead to energy cost concentration. In DMRF + LEACH, although it can handle the energy concentration problem, the energy difference between the maximum and the minimum is still high, whereas in GTRF, the node with the highest energy is selected as the cluster head, which balances the energy cost a lot. Therefore, GTRF outperforms the other two methods in terms of energy difference. Compared with Figure 9, the selfish nodes do not influence the energy differences much in DMRF or GTRF. Only slight changes exist in DMRF + LEACH. Therefore, only a few distinctions occur between Figure 8 and Figure 9. We can conclude that GTRF can balance the energy cost.

### 5.2. Network Lifetime 

**Figure 10:**
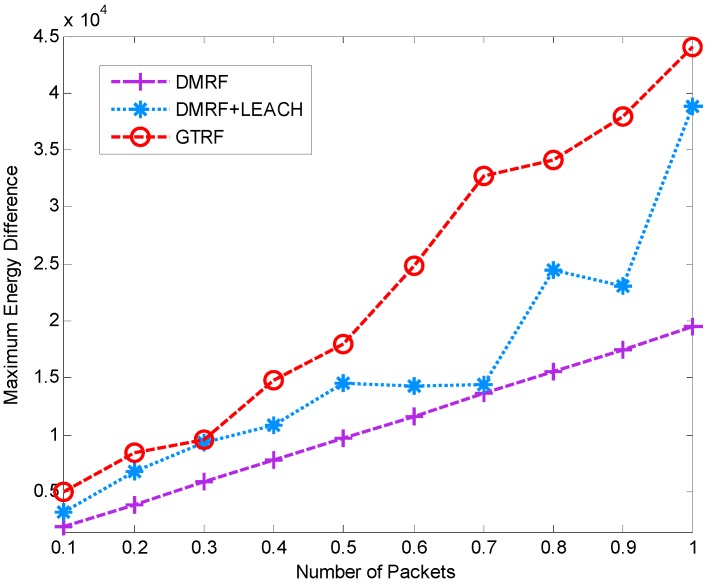
Lifetime of the network with selfish nodes.

**Figure 11:**
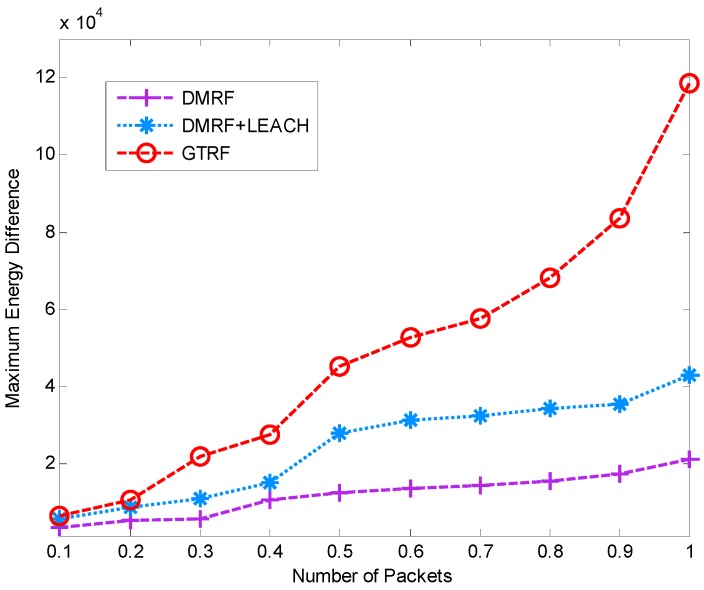
Lifetime of the network without selfish nodes.

In this section, the lifetime of the network is shown in Figure 10 and Figure 11. We measure how long a network will sustain until any node exhausts. In Figure 10, with the increment of the initial energy of nodes, each method presents an increasing tendency in lifetime. Only a slow growth appears in DMRF as well as DMRF + LEACH. In DMRF, a small amount of nodes always exist in others’ forwarding candidate sets. These nodes will firstly run out of power. In DMRF + LEACH, compared with DMRF, although the network doubles in lifetime, issue of unequal size of clusters energy is still unsolved. Consequently, the lifetime of the network is directly relevant to cluster with the smallest size. Whereas in GTRF, by implementing FATP protocol [26], VA generates clusters with nearly equal sizes and guarantees the balance of energy cost, therefore, it can prolong the lifetime a lot.

Compared with Figure 11, distinctions appear in the lifetime of each approach, which results from the effect of the selfish nodes. Since in DMRF and DMRF + LEACH, the selfish nodes are regarded as faulty nodes which cast a burden on other nodes, therefore, the selfish nodes can shorten the lifetime of the network indirectly. From Figure 10 and Figure 11, we can conclude that GTRF can prolong the lifetime of the network. 

### 5.3. Control Packets

In this section, we analyze the number of control packets in the network. In GTRF, the control messages are especially used in three conditions:

(1) The cluster head periodically sends the control packets to check the validity of each cluster member.
(2) In the forwarding process, when the void region is detected, the feedback packets will be sent to inform the upstream node that there are selfish nodes located downstream.
(3) When the sink node receives the packets, it will notify the source that the packets have been successfully received by the sink.

**Figure 12:**
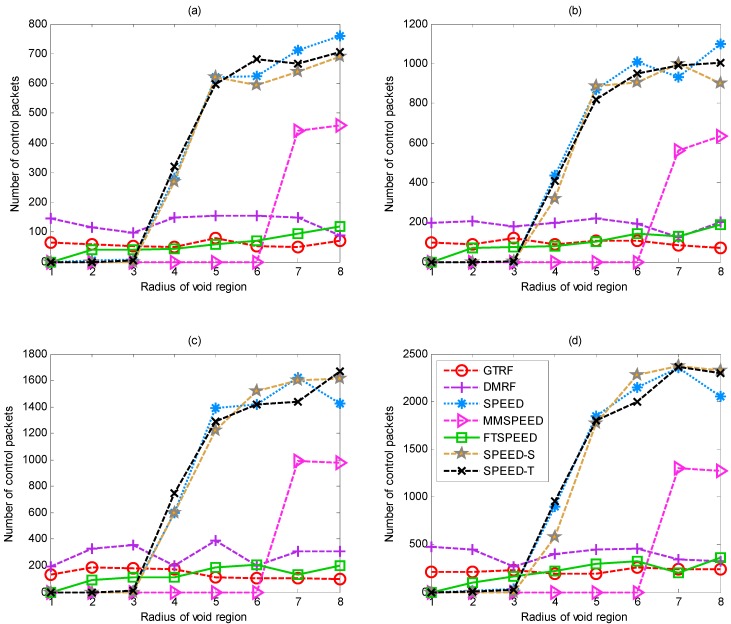
Number of control packets after transmitting (**a**) 1000; (**b**) 2000; (**c**) 5000 and (**d**) 10,000 packets under different routing protocols.

In Figure 12, we analyze the relationship between the radius of void region and number of control packets. We record the number of control packets by transmitting 1000, 2000, 5000 and 10,000 packets, respectively. All the selfish nodes will be considered as faulty nodes in all the protocols except GTRF. Due to the increasing size of the void region (the radius of the void region is within [0,5]), packet loss appears frequently, which increases the number of controlling packets in SPEED, SPEED-T, SPEED-S and MMSPEED. Whereas in FTSPEED, DMRF and GTRF, the number of the control packets changes relatively smooth. GTRF is slightly lower than DMRF and FTSPEED, because the GTRF utilizes the cluster structure; only the cluster head is capable of sending the control messages. When the radius exceeds above five, the result of each protocol displays a slow growth tendency which results from the fewer nodes working in the network. Since GTRF utilizes a cluster structure combined with the jumping transmission manner, the void region does not affect GTRF much, so the number thereby is not perturbed a lot. 

### 5.4. Delay of Transmission

**Figure 13:**
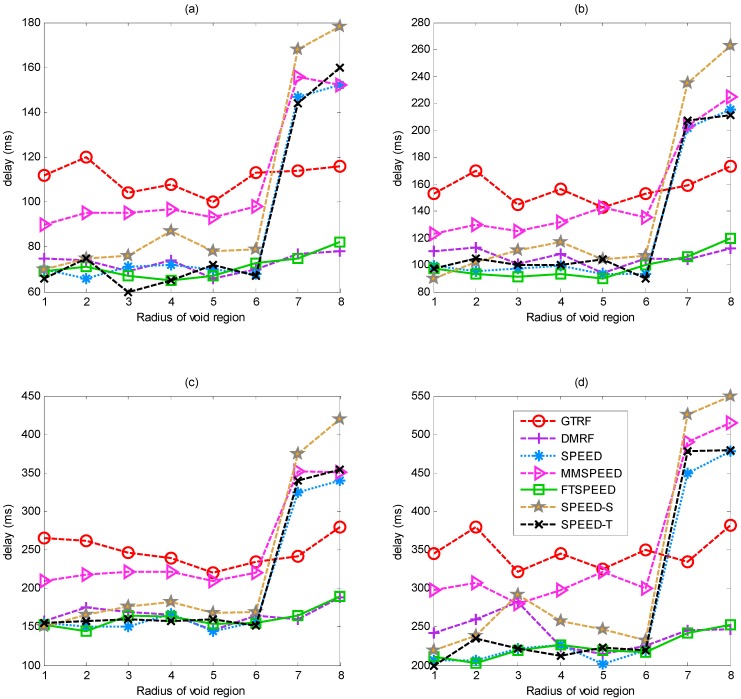
Comparison of transmission delay after transmitting (**a**) 1000; (**b**) 2000; (**c**) 5000 and (**d**) 10,000 packets under different routing protocols.

The average data transmission delay with respect to different void region radii is shown in Figure 13. Owing to the data fusion delay in clusters, GTRF spends more time than the hop-by-hop routing protocol. However, GTRF still has the merit that fewer nodes will appear within a path, which reduces the possibility of encountering a void region when forwarding packets. Therefore, the probability of such a delay is small. Figure 13 indicates that there is no direct relationship between the size of the void region and the network delay in GTRF. For transmitting 1000, 2000, 5000 and 10,000 packets, GTRF presents nearly the same transmission delay tendency. Although the delay of GTRF is higher than FTSPEED and DMRF, and even in some cases, it is also higher than the other real-time routing protocols (e.g., SPEED, SPEED-T, SPEED-S, MMSPEED), GTRF still can achieve real-time transmission.

## 6. Conclusions and Future Works

In this paper, a game theory-based real-time fault-tolerant routing protocol called GTRF has been proposed. GTRF consists of two parts: a VA model and a jumping transmission model. In the VA model, we design the auction process and some rules for nodes to obey. We proved that the auction situation the satisfies Nash Equilibrium if every node bids its actual energy. Then the jumping transmission model is proposed which applies a jumping manner when a void region is detected or the remaining time ratio of a message falls below a threshold. We also prove that GTRF can meet real-time requirements. Finally, extensive simulations are conducted to show the advantages of GTRF. These simulation results show that GTRF can not only efficiently balance the energy cost of the network, prolonging the lifetime, but also utilize fewer control packets in achieving real-time transmission. As part of our future work, we would like to extend GTRF in the data aggregation area. It is also valuable to research a multi-level VA model, which can not only further conserve energy, but also relieve the burden of the sink nodes. 
